# Comprehensive Utilization Technology of *Aronia melanocarpa*

**DOI:** 10.3390/molecules29061388

**Published:** 2024-03-20

**Authors:** Dongfang Shi, Jing Xu, Li Sheng, Kai Song

**Affiliations:** 1 Institute of Innovation Science and Technology, Changchun Normal University, Changchun 130032, China; shidongfang@ccsfu.edu.cn (D.S.); xujingl@yeah.net (J.X.); 2 School of Life Science, Changchun Normal University, Changchun 130032, China; 3 Jilin Qiu Zhiyuan Ecological Technology Co., Ltd., Siping 136000, China

**Keywords:** *Aronia melanocarpa*, utilization, active ingredients, physiological functions, processing technology

## Abstract

*Aronia melanocarpa* fruit contains a variety of active ingredients, such as phenolic acids, anthocyanins, proanthocyanidins, etc. Relevant in vivo and in vitro studies have concluded that it has beneficial effects in terms of treating dyslipidemia, hypertension, glucose metabolism disorders, etc. This article discusses the nutritional value and food processing of *Aronia melanocarpa* and reviews the chemical components of *Aronia melanocarpa* and the pharmacological activities of related substances in order to summarize the chemical characteristics of the fruit and its development prospects. The process optimization of juice production, the impact of antioxidant capacity, and the comprehensive utilization of pomace in feed are discussed. This article provides a reference for future comprehensive application research and product development of *Aronia melanocarpa*.

## 1. Introduction

*Aronia melanocarpa*, also known as black heather and commonly known as the eternal berry, belongs to the family Rosaceae and is a deciduous shrub. Originating in North America, it slowly expanded to Europe through variety selection and introduction and through cultivation. It has been cultivated around the world for more than 100 years. At present, it has established a certain scale in terms of cultivation area and related supporting processing industries [1,2]. It was introduced to China in the 1990s and is currently grown in Liaoning, Jilin, Heilongjiang, Shandong, Jiangsu, Anhui, and other provinces [3,4]. There are more than 30 global varieties of *Aronia melanocarpa*, which are mainly divided into diploids selected in North America and polyploids bred in Europe. The *Aronia melanocarpa* plant has strong resistance to diseases and insect pests. It has dense and colorful bouquets, a long flowering period, and red leaves in autumn. Therefore, it is also widely used in gardens in Europe, America, and East Asia [5].

The fruit of *Aronia melanocarpa* is purple–black in color and contains dietary fiber, protein, sugars, polyphenols, and other nutrients. Of these, polyphenols are its main active ingredients, including proanthocyanidins, flavonoids, and phenolic acids [6,7]. *Aronia melanocarpa* is known for its high content of anthocyanin, a class of powerful antioxidants. Aronia generally contains a higher proportion of anthocyanins than other fruits of the Rosaceae family [8], especially compared to strawberries, apples, or pears, which makes it even more prominent in its antioxidant capacity. In addition to anthocyanins, Aronia is rich in other polyphenolic compounds such as proanthocyanidins and flavonoids [9]. While these compounds are found in many fruits of the rose family, the content and variety are generally more abundant in Aronia.

Due to its high content of anthocyanins and other polyphenolic compounds, Aronia exhibits greater antioxidant capacity than many other fruits of the Rosaceae family [10]. This helps reduce oxidative stress and the risk of chronic disease. Aronia’s bioactive compounds help reduce the risk of cardiovascular disease by improving blood pressure, reducing inflammation, and reducing arteriosclerosis [11]. While other Rosaceae fruits, such as apples and cherries, have also shown cardiovascular protective effects, Aronia has been studied more in this area, and its effects may be more significant. Compounds in Aronia help regulate blood sugar and improve insulin sensitivity, possibly more than other fruits in the rose family.

Due to its powerful health benefits, Aronia is widely used to make nutritional supplements and health drinks; perhaps more commonly than other fruits of the Rosaceae family [12]. Although many fruits of the Rosaceae family are used in jams, beverages, and desserts, Aronia has unique uses as food coloring and natural additive due to its special taste and color (dark purple to black) [13].

Although *Aronia melanocarpa* has unique advantages in terms of composition, bioactivity, and uses, consumer acceptance and market penetration are still limited by its taste (which may be astringent) and popularity in certain regions. In the future, as people’s interest in healthy foods increases, the popularity of Aronia and its products is likely to increase further.

Currently, it is mainly used in the food and pharmaceutical fields [14]. The development of *Aronia melanocarpa* resources is currently mainly focused on fruits. Recent studies have found that *Aronia melanocarpa* and its extracts not only have a significant improvement effect on common diseases such as dyslipidemia, hypertension, obesity, glucose metabolism disorders, and thrombosis [15,16], but it can also inhibit the proliferation of breast cancer, pancreatic cancer, and intestinal cancer tumor cells [14,17,18].

*Aronia melanocarpa* fruit is berry-like when mature and has a high juice yield. It contains high quantities of tannins, and these tannins are responsible for the astringent and slightly bitter taste of the fruit, causing the *Aronia melanocarpa* fruit to taste sour and have a poor taste. Therefore, it can be made into juice, which can better retain the original flavor and nutrients while making the taste better and easier for consumers to accept [19]. However, *Aronia melanocarpa* juice is rich in unbroken cells, pectin, cellulose, hemicellulose, and other macromolecules (anthocyanins, proanthocyanidins, tannins, and flavonoids). The complex components of the system are prone to flocculation, sinking, and stratification which, in turn, affect the color, flavor, and taste of the juice, seriously affecting the product value. Therefore, it must be handled during processing.

On the one hand, one of the most important factors in the juice extraction process on an industrial scale is to obtain the highest possible extraction efficiency. On the other hand, for consumers, the most important quality indicators when purchasing the juice are its health-promoting properties and sensory qualities. Therefore, when selecting the process conditions for the production process, these two conditions should be met at the same time. According to different processing techniques, the juice is divided into clear juice and cloudy juice. Clear juices are mostly treated with clarifying agents such as gelatin, chitosan, pectinase, and cellulase. For the cloudy juice, the homogenization method must be used.

So far, research on *Aronia melanocarpa* has mainly focused on the extraction of active ingredients from the fruit and antioxidant research, with less research on the process optimization and antioxidant capacity of its products. With the development of the *Aronia melanocarpa* food industry, related companies will produce a large amount of *Aronia melanocarpa* pomace, which is still rich in polymerized proanthocyanidins, anthocyanins, lignin, and cellulose. Failure to properly dispose of *Aronia melanocarpa* pomace will lead to the waste of a large amount of high-value resources and increase the cost of wet waste disposal for society. It is of great practical significance to explore how to utilize *Aronia melanocarpa* pomace reasonably and efficiently.

Therefore, this article aims to deepen our understanding of the characteristics of the fruit’s nutritional components and active ingredients by reviewing relevant research on the chemical components and activities in *Aronia melanocarpa* fruit. The effects of different processing methods on the quality and antioxidant activity of *Aronia melanocarpa* juice are discussed. By reviewing the current utilization status of *Aronia melanocarpa* pomace in feed, we hope to provide reference and theoretical support for the comprehensive utilization of *Aronia melanocarpa* fruit resources.

## 2. *Aronia melanocarpa’s* Active Ingredients

**Figure 1:**
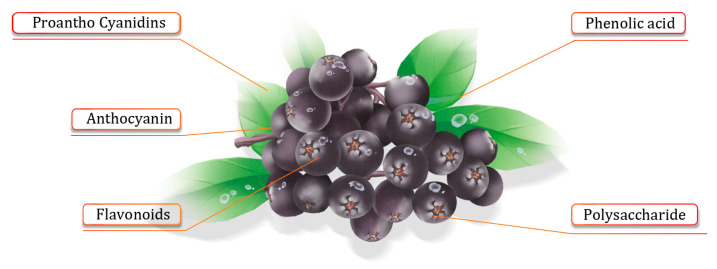
*Aronia melanocarpa’s* active ingredients.

Due to the high content of tannins and other polyphenolic compounds in *Aronia melanocarpa*, the fruit tastes astringent and is rarely eaten directly. It is often further processed to produce juice, jam, fruit tea, and fruit wine and to extract natural food pigments. In addition, *Aronia melanocarpa* is rich in vitamins (vitamins B2, B3, and B9), minerals (copper, manganese, molybdenum, and trace amounts of iron, zinc, and cobalt), polysaccharides, and other conventional ingredients. The fact that it also contains polyphenolic compounds, such as proanthocyanidins, quercetin, flavonoids, and phenolic acids, as shown in Figure 1, is worthy of further attention. Currently, *Aronia melanocarpa* is recognized as one of the richest plant sources of phenolic bioactive substances.

### 2.1. Proanthocyanidins

Proanthocyanidins are the main phenolic active substances in *Aronia melanocarpa* fruit (as shown in Table 1). Proanthocyanidins are mainly composed of polymers formed by different numbers of catechin units (including epicatechin) connected through C-C bonds. This is also the main reason for the bitter taste of the fruit [20]. Proanthocyanidins exist in the form of monomers (0.78%), dimers (1.88%), trimers (1.55%), 4–6-mers (6.07%), 7–10-mers (7.96%), and those of more than 10 polymers (81.72%) [21]. Zhu et al. [22,23] used response surface methodology to optimize the extraction process of proanthocyanidins and obtained a proanthocyanidin content of 3.32% in *Aronia melanocarpa*. Then, macroporous resin was used for purification, and the contents of proanthocyanidin B2 and epicatechin in the purified product were measured as 22.45 and 14.26 mg/g, respectively. Zhang et al. [24] used ultra-high-performance liquid chromatography technology to measure the proanthocyanidin B1, proanthocyanidin B2, and proanthocyanidin B4 contents of *Aronia melanocarpa* as 129.78, 3834.99, and 196.02 μg/g, respectively.

### 2.2. Anthocyanins

Meng et al. [27] tested the anthocyanin components of *Aronia melanocarpa*. The main ones found were *cyanidin-3,5-dihexoside*, *cyanidin-hexoside dimer*, *cyanidin-3-O-(galactoside, glucoside, arabinoside, xyloside)*, and *delphinidin-3-O-rutinoside dimer*. Zhi et al. [28] used response surface methodology to determine the optimal extraction process of anthocyanins. At a temperature of 50 °C, a time of 1.2 h, a methanol volume fraction of 60%, and a solid-to-liquid ratio of 1:5 (g/mL), the anthocyanin extraction rate was as high as 0.31%, and the anthocyanin purity was 22%. Research by Gao et al. [29] showed that the main substance of *Aronia melanocarpa* polyphenols is highly polymerized proanthocyanidin. This can be directly converted into cyanidin under heat–acid–alcohol treatment. After conversion treatment, the antioxidant activity of highly polymerized proanthocyanidin on cells is increased 4.77 times.

### 2.3. Flavonoids

Flavonoids are one of the important active ingredients of *Aronia melanocarpa*. Li et al. [30] used gas chromatography–mass spectrometry (GC-MS) technology to detect flavonoids and found that the average content of total flavonoids in *Aronia melanocarpa* was 0.233%. Liu et al. further improved the extraction rate of flavonoid compounds from Rowan nigra through orthogonal experiments. In addition, studies by relevant scholars have shown that *Aronia melanocarpa* flavonoids include quercetin, rutin, hyperoside, etc. Song et al. [31] used ionic liquid ultrasound-assisted technology to extract hyperin and found that its content was 27.37 μg/g.

### 2.4. Phenolic Acids

Phenolic acid compounds refers to a class of compounds with several phenolic hydroxyl groups on the same benzene ring. This class includes caffeic acid, chlorogenic acid, neochlorogenic acid, gallic acid, gallic acid, protocatechuic acid, ferulic acid, sinapinic acid, etc. The main components of phenolic acids in *Aronia melanocarpa* are chlorogenic acid and neochlorogenic acid [32]. Ochmian et al. [33] measured the chlorogenic acid content of 100 g of *Aronia melanocarpa* to be 72–84 mg and the neochlorogenic acid content to be 62.2–72.1 mg. Wei et al. [34] used high-performance liquid chromatography (HPLC) to detect phenolic acids in *Aronia melanocarpa* extract. Mainly the following phenolic acids were found: 0.045 mg/g of gallic acid, 0.102 mg/g of protocatechuic acid, 0.055 mg/g of p-hydroxybenzoic acid, 0.686 mg/g of caffeic acid, 10.206 mg/g of benzoic acid, 0.295 mg/g of p-coumaric acid, 0.267 mg/g of ferulic acid, and 0.253 mg/g of cinnamic acid.

### 2.5. Polysaccharides

Su et al. [35] optimized the extraction process of *Aronia melanocarpa* polysaccharides through response surface methodology. Under the conditions of a solid-to-liquid ratio of 1:10 (g/mL), a microwave power of 500 W, and an extraction time of 25 min, the polysaccharide extraction rate was as high as 4.46%. Wei et al. [36] used the microwave-assisted method to extract *Aronia melanocarpa* polysaccharides, and the extraction rate reached 4.44%. Qi et al. [37] used UV–visible spectrophotometry and high-performance liquid chromatography to determine the polysaccharide content of wild blueberries, viburnum, hawthorn, mountain vitex, and *Aronia melanocarpa*. The results are shown in Table 2. The polysaccharide content of *Aronia melanocarpa* is higher than that of wild blueberries, viburnum, and hawthorn. Yao et al. [38] used UV–visible spectrophotometry to determine the amount of polysaccharides in *Aronia melanocarpa*. It was found that the polysaccharide content of *Aronia melanocarpa* was higher than that of the small northern berry known as indigo.

## 3. *Aronia melanocarpa’s* Physiological Functions

### 3.1. Antioxidant Properties

*Aronia melanocarpa* extract has strong antioxidant activity. Gao et al. [39] used the polyphenols in *Aronia melanocarpa* extract to conduct in vitro antioxidant tests. The results show that *Aronia melanocarpa* polyphenols have the ability to scavenge 1,1-diphenyl-2-picrylhydrazyl (DPPH) free radicals, 2,2′-azinobis(3-ethylbenzothiazoline-6-sulfonate) free radicals, and superoxide anion free radicals. Furthermore, as the concentration increases, the antioxidant activity of *Aronia melanocarpa* polyphenols increases, and its antioxidant capacity is even better than that of vitamin C, as shown in Table 3.

**Figure 2:**
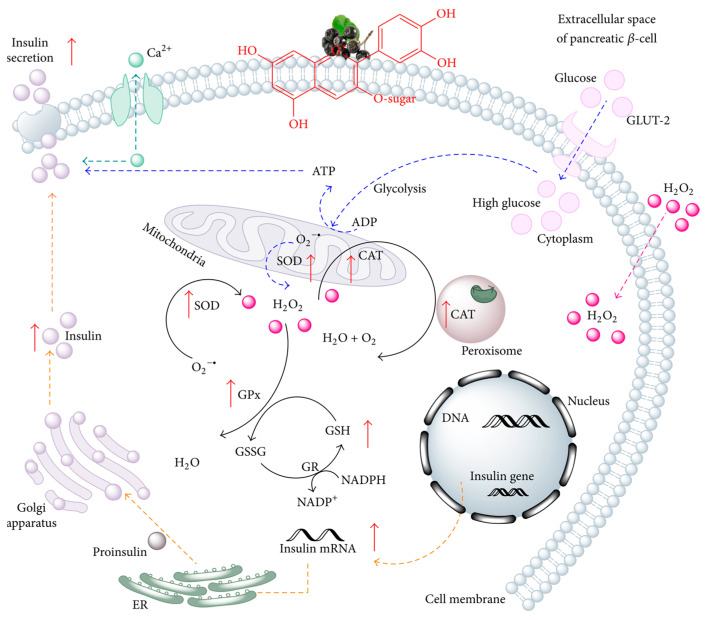
Antioxidant effects of anthocyanins on pancreatic β-cells (βTC3) exposed to oxidative stress conditions induced by hydrogen peroxide (H_2_O_2_^−^) and high glucose (HG^−^) [41]. The red arrow indicates that the relative reactivity increases to varying degrees.

Marie et al. [20] have shown that cyanidin-3-galactoside, cyanidin-3-glucoside, and cyanidin-3-arabinoside extracted from *Aronia melanocarpa* have a very strong cleaning ability on DPPH free radicals. In addition, Huang et al. [40] found that the extract from the seeded fruit of *Aronia melanocarpa* has stronger in vitro antioxidant capacity than the seeded fruit of other berries, which may be related to its polyphenol content. Dumitriţa et al. [41] showed, through animal experiments, that *Aronia melanocarpa* polyphenols can effectively scavenge superoxide anions produced by ionizing radiation. Anthocyanins may increase insulin gene expression as chokeberry anthocyanin extract (CAE) treatment attempts to restore the insulin pool, as shown in Figure 2. Li et al. [42] fed mice 3% *Aronia melanocarpa* juice for 8 consecutive weeks and found that the superoxide dismutase (SOD) activity in the liver and kidneys of the mice increased. The content of malondialdehyde (MDA) decreased significantly, and the content of glutathione (GSH) increased. This shows that *Aronia melanocarpa* extract has strong antioxidant activity in the body.

### 3.2. Antibacterial Properties

Zhu et al. [22] conducted antibacterial tests on *Escherichia coli*, *Staphylococcus aureus*, *Bacillus subtilis*, and *Saccharomyces cerevisiae*. The results show that *Aronia melanocarpa* proanthocyanidins have strong inhibitory effects on bacteria such as *Bacillus subtilis*, *Staphylococcus aureus*, and *Escherichia coli*, but have almost no effect on *Saccharomyces cerevisiae*. The proanthocyanidins, before purification, have stronger antibacterial ability than the proanthocyanidins after purification. In addition, Miao et al. [43] found that *Aronia melanocarpa* polyphenols have significant inhibitory effects on five types of bacteria, including *Bacillus subtilis*, *Alicyclobacillus*, *Enterobacter cloacae*, *Enterobacter aerogenes*, and *Saccharomyces cerevisiae*. Denev et al. [44] conducted antibacterial tests on different polyphenols extracted from *Aronia melanocarpa* and showed that *anthocyanins*, *proanthocyanidins*, *neochlorogenic acid*, and *chlorogenic acid* were effective against *Escherichia coli*, *Staphylococcus aureus*, *Salmonella enterica*, *Listeria monocytogenes*, and 10 kinds of pathogenic bacteria, including *Proteus*, *Pseudomonas aeruginosa*, and *Candida albicans*, and have certain antibacterial effects. *Aronia melanocarpa* procyanine was found to have the strongest antibacterial effect. Marie et al. [45] confirmed that cyanidin-3-galactopyranoside, cyanidin-3-glucoside, and proanthocyanidins in *Aronia melanocarpa* can inhibit the formation of Escherichia coli and Bacillus cereus biofilms, thereby achieving antibacterial effects.

### 3.3. Anti-Inflammatory Properties

**Figure 3:**
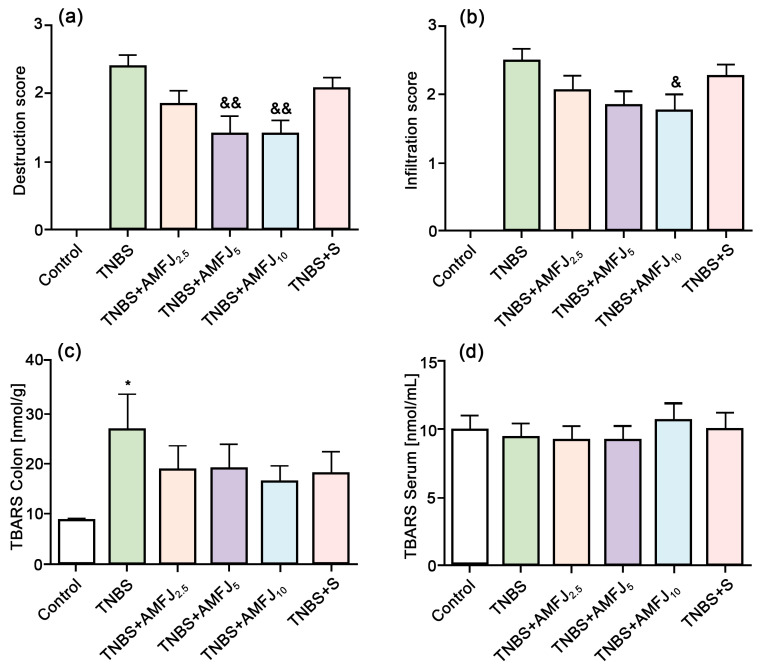
Effects of *Aronia melanocarpa* extract and sulfasalazine (S) on thiobarbituric acid reactive substance (*TBARS*) levels in rats: (**a**) epithelium and glands, (**b**) inflammatory cells, colon (**c**), and serum (**d**) [47]. The && in Figure 3a indicated that compared with the TNBS group, the epithelial and glandular destruction scores of rats in TNBS+ amfj5 and TNBS+ amfj10 groups were significantly lower than those in the TNBS group (*p* < 0.01), with statistical significance. As shown in Figure 3b &, compared with TNBS group, the inflammatory cell infiltration scores of rats in TNBS+ amfj5 and TNBS+ amfj10 groups were lower than those in TNBS group, but TNBS+ amfj10 group was more statistically significant (compared with TNBS group, TNBS+ amfj10 group, *p* < 0.05). * in Figure 3c indicates that in the colons of rats in the TNBS group, TNBS caused oxidative stress in the colons of rats, increased lipid peroxidation, and the concentration of TBARS was significantly higher than that of the control group (*p* < 0.05).

Bararu et al. [46] fed diabetic rats *Aronia melanocarpa* extract and found that the number of monocytes and granulocytes that cause inflammation in the rats was reduced, and the number of lymphocytes that cause atherosclerotic plaque formation was inhibited. This shows that it has a strong anti-inflammatory effect in the body. Kuzmanova et al. [47] used trinitrobenzene sulfonic acid to induce colitis in rat models and then fed them 2.5, 5.0, or 10.0 mL/kg *Aronia melanocarpa* extract and 400 mg/kg sulfasalazine for 14 consecutive days. The study found that the anti-inflammatory effect of 2.5 or 5.0 mL/kg of Sorbus nigra extract was equivalent to that of the positive control sulfasalazine, as shown in Figure 3. Zapolska-Downar et al. [48] also confirmed that *Aronia melanocarpa* extract has a strong anti-inflammatory effect. It inhibits the transcription of intercellular adhesion molecule-1 (*ICAM-1*) and vascular cell adhesion molecule-1 (*VCAM-1*) genes mediated by the tumor necrosis factor alpha (*TNF-α*) signaling pathway and reduces the expression of adhesion molecules in aortic endothelial cells (*HAECs*). In addition, Bijak et al. [49] have shown that *Aronia melanocarpa* extract has a certain protective effect on plasma fibrinogen damage caused by peroxynitrite ions. Therefore, it can be used to regulate peroxynitrite formation and reduce the risk of inducing inflammation.

### 3.4. Inhibiting Tumor Cell Growth

Through in vitro studies, Cvetanović et al. [50] showed that *Aronia melanocarpa* extract has a strong inhibitory effect on HeLa cervical cancer cells. Its *IC50* value is 5.44 μg/mL, which is much lower than the *IC50* value of 7.46 μg/mL of the anticancer drug cisplatin. Experiments have found that *Aronia melanocarpa* extract also has a strong inhibitory effect on LS-174T human colorectal adenocarcinoma cells. Rugină et al. [51] used *Aronia melanocarpa* anthocyanins at 100 and 200 μg/mL to reduce the in vitro survival rate of HeLa cervical cancer cells to 30% and 40%, respectively. ROS levels in HeLa cells increased significantly, proving that *Aronia melanocarpa* anthocyanins can inhibit the growth of HeLa cervical cancer cells. Choi et al. [52] fermented *Aronia melanocarpa* juice with Lactobacillus rhamnosus and produced catechins that inhibited the growth of *MCF7* and *MDA-MB-231* breast cancer cells, thereby reducing the expression of *Nanog*, *Sox2*, and *OCT4* genes in breast cancer stem cells and inhibiting the proliferation of breast cancer cells.

**Figure 4:**
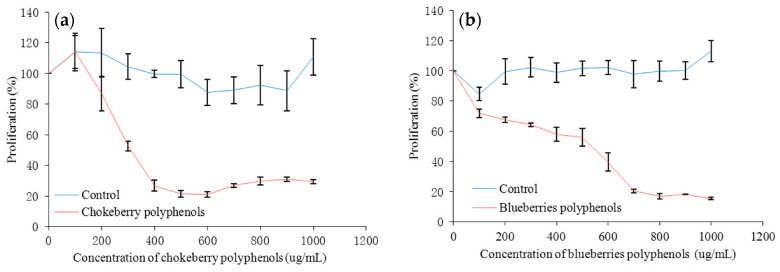
Inhibitory effect of chokeberry polyphenol extract on the proliferation of human liver cancer cell *HepG2*. (**a**) *Aronia melanocarpa*; (**b**) blueberry [53].

Gao et al. [53] have shown that *Aronia melanocarpa* extract can inhibit the proliferation of the human liver cancer cell *HepG2*, as shown in Figure 4. *Aronia melanocarpa* polyphenols below 350 μg/mL are not toxic to cells, indicating that the inhibition of proliferation is not caused by cytotoxic effects. Thi et al. [54] found that *Aronia melanocarpa* extract had a significant inhibitory effect on the human liver cancer cell *SK-Hep1*. In the mass concentration range of 0–400 μg/mL, its inhibitory rate on cell proliferation ranges from 5.5% to 48.8%. *Aronia melanocarpa* extract inhibits the expression of *MMP-2* and *MMP-9*, thereby causing *G2/M* phase cell cycle arrest and reducing the expression of the antiapoptotic *Bcl-2* gene.

Cigarette smoke contains large amounts of highly carcinogenic compounds. Balansky et al. [55] found that short-term exposure of mice to smoke can cause histopathological changes in the red blood cells, lungs, liver, and bladder in mice. Feeding mice *Aronia melanocarpa* extract can reduce the formation of lung adenomas, emphysema, and liver degeneration caused by cigarette smoke. *Aronia melanocarpa* extract was significantly more effective in female mice. Lung cancer and breast cancer rank first among malignant tumors in men and women in China, and the incidence of digestive tract tumors is increasing year by year. It can be seen that the potential of *Aronia melanocarpa* in cancer prevention and treatment is unlimited.

### 3.5. Lowering Blood Pressure

Kardum et al. [56] prepared a dietary supplement of *Aronia melanocarpa* juice and glucomannan. Then, a one-month experiment was conducted on 20 obese women aged 45 to 65 years old. The subjects were required to ingest 100 mL of supplement daily. The results showed that these volunteers’ systolic blood pressure (SBP) decreased from an average of 17.0 kPa to an average of 15.5 kPa. Another study by Kardum et al. [57] found that hypertensive adults (33–67 years old) who had not received hypertension or class I hypertension medication and ingested 200 mL of *Aronia melanocarpa* juice daily for 4 weeks showed significant reductions in both systolic and diastolic blood pressure. In addition, triacylglycerol, total cholesterol, and low-density lipoprotein were significantly reduced. This shows that *Aronia melanocarpa* has certain blood-pressure-lowering effects on obese and hypertensive people. Loo et al. [58] conducted an 8-week trial on 38 patients with hypertension and found that daily intake of 300 mL of *Aronia melanocarpa* juice and 3 g of *Aronia melanocarpa* fruit powder could reduce diastolic blood pressure in patients with hypertension. In addition, Kardum et al. [59] found that healthy adult women who consumed 100 mL of *Aronia melanocarpa* juice daily for 3 months had no significant change in systolic blood pressure. This shows that *Aronia melanocarpa* juice has little effect on blood pressure in healthy people.

### 3.6. Lowering Blood Sugar

Lipińska et al. [60] used 24 Polish Merino lambs as experimental animals. After continuous feeding for 90 days, the test showed that the glucose level of the lambs in the control group was 3.38 mmol/L, and, in the lambs fed 150 or 300 g/kg *Aronia melanocarpa* pomace feed, glucose levels were 2.42 and 1.55 mmol/L, respectively. This shows that *Aronia melanocarpa* pomace can induce a hypoglycemic effect in lambs. Qin et al. [61] used a high-fructose diet to induce metabolic disorder in rat models; then, the rat models were fed *Aronia melanocarpa* extract at a dose of 100 or 200 mg/kg body weight. They found that the blood sugar levels of the rats were significantly reduced. Takahashi et al. [62] found that *Aronia melanocarpa* extract reduced the blood sugar level of rats fed high-sugar and high-fat diets. Experiments proved that *Aronia melanocarpa* could reduce the blood sugar of rats to 7.0 mmol/L. In addition, Park et al. [63] found that *Aronia melanocarpa* extract could reduce blood sugar in insulin-resistant rats.

Worsztynowicz et al. [64] found that chlorogenic acid and cyanidin-3-glucoside, the active ingredients of *Aronia melanocarpa*, can significantly inhibit α-amylase activity. A study by Marie et al. [20] concluded that the *EC50* of cyanidin-3-arabinoside, cyanidin-3-glucoside, and cyanidin-3-galactoside in *Aronia melanocarpa*, for α-glucosidase, was 0.37, 0.87, and 1.54 μg/mL, respectively. This shows that these anthocyanins have a strong inhibitory effect on α-glucosidase activity, thereby lowering blood sugar. Dipeptidyl peptidase 4 (DPP-4) inhibitors are commonly used drugs to treat diabetes. They inhibit the decomposition of glucagon-like peptide-1 (*GLP-1*) and glucose-dependent insulinotropic polypeptide (*GIP*), thereby increasing the levels of endogenous *GLP-1* and *GIP* and controlling blood sugar. Kozuka et al. [65] found that the inhibitory effect of *Aronia melanocarpa* extract on *DPP-4* activity was approximately 27%. Further research revealed that the chemicals that inhibit *DPP-4* activity are cyanidin-3-glucoside and cyanidin-3,5-dihexoside. In addition, it was found that the effect of cyanidin-3,5-dihexoside was more significant. Therefore, it is believed that *Aronia melanocarpa* can be used as a potential source of hypoglycemic drugs.

### 3.7. Preventing the Toxic Effects of Various Substances

Experiments conducted by Borowska et al. [66] showed that *Aronia melanocarpa* products can effectively prevent and treat diseases caused by oxidative stress induced by heavy metals or toxic substances. Kondeva-Burdina et al. [67] exposed rat liver cells to CCl_4_ and tert-butyl hydroperoxide (tBuOOH). It was found that *Aronia melanocarpa* juice can protect liver cells by improving cell viability, increasing glutathione (*GSH*) concentration, and reducing malondialdehyde (*MDA*) and lactate dehydrogenase (*LDH*) concentrations. In addition, its effect was better than that of the positive control silymarin.

Kuzmanova et al. [68] found that *Aronia melanocarpa* juice can inhibit the hepatotoxic effects caused by taking paracetamol and protect the liver, but its mechanism of action has not been reported. Paraquat induces apoptosis by increasing ROS production, leading to neurotoxic health disorders. Case et al. [69] conducted a study on the inhibitory effect of *Aronia melanocarpa* extract on neurotoxicity caused by paraquat. *Aronia melanocarpa* extract was found to inhibit paraquat-induced oxidative stress and apoptosis in *NG108-15* cells. Brzóska et al. [70] have shown that *Aronia melanocarpa*, when ingested by rats, can inhibit bone metabolism abnormalities caused by cadmium (CdCl_2_· 5H_2_O).

Antonisamy et al. [71] conducted a study on the effect of *Aronia melanocarpa* extract on ethanol-induced gastric ulcers in rats. The results showed that *Aronia melanocarpa* extract significantly reduced gastric mucosal damage and reduced edema formation. A reduction in the levels of inflammatory markers, modulation of *HSP-70* protein, *NF-κB* protein, and *MCP-1* protein, as well as a significant improvement in the antioxidant capacity of the rat body were observed.

### 3.8. Antidepressant Properties

Eftimov et al. [72] studied the effect of *Aronia melanocarpa* juice on the behavior of socially isolated male Wistar rats. The results showed that social isolation caused a decrease in the exercise ability of rats in the field test, but feeding the rats *Aronia melanocarpa* juice had no significant effect. The results of the social interaction test and forced swimming test showed that social isolation led to an increase in the immobility time of rats. *Aronia melanocarpa* juice clearly prevented this behavior from occurring, indicating that *Aronia melanocarpa* juice has a certain antidepressant effect. Tomić et al. [73] found that male rats fed *Aronia melanocarpa* juice showed a significantly reduction in hyperactivity in field tests and in anxiety-like behaviors in the elevated plus maze. In the forced swimming test, it was found that the group of rats that were administered juice showed a reduction in depressive-like behaviors, indicating *Aronia melanocarpa*’s potential as an antidepressant.

### 3.9. Improving Memory

Kuzmanova et al. [74] used 224 male Wistar rats as experimental animals. There were 14 rats in each group. The rats were orally administered 2.5, 5.0, or 10.0 mL/kg of *Aronia melanocarpa* juice. At the same time, 10 mL/kg physiological saline was used as a blank control and fed for 7, 14, 21, or 30 days, respectively. Memory assessment was then performed using the shuttle passive avoidance test. The results showed that the incubation period and learning standard of oral administration of *Aronia melanocarpa* juice for 7 and 14 days were dose-dependent, but the effect was not significant. After 21 d of treatment, juice dose dependently prolonged the latency of the retention test. The effect of 5 or 10 mL/kg dose was more significant. After 30 days of oral administration, each test dose of juice significantly increased the latency of the retention test. The 10 mL/kg dose significantly increased the percentage of rats reaching learning criterion. This shows that *Aronia melanocarpa* juice can improve the memory and enhance the learning ability of rats.

### 3.10. Anti-Fatigue Properties

Li [75] used *Aronia melanocarpa* flavonoids to conduct a 30-day gavage test on mice. The results showed that the swimming time of mice was significantly prolonged, and the lactic acid content in the exercising mice decreased significantly. This shows that *Aronia melanocarpa* has a certain anti-fatigue effect.

### 3.11. Other Functions

*Aronia melanocarpa* extract can directly or indirectly inhibit viral replication and has the potential to prevent and treat influenza [76]. Park et al. [77] have shown that *Aronia melanocarpa* polyphenols have in vivo inhibitory effects on various subtypes of influenza viruses, such as *H_1_N_1_*, *H_3_N_2_*, and *H_5_N_1_*, and can protect mice from fatal virus attacks. Zhang et al. [78] showed that 125 μg/mL anthocyanins can significantly reduce the ROS content and *MMP-1* secretion level of cells after UVA radiation damage of 10 J/cm^2^, indicating that *Aronia melanocarpa* anthocyanins have the effect of reducing UV damage.

**Table 3:** *Aronia melanocarpa*’s main bioactive components and physiological functions and their mechanisms of action [13,79,80,81].

Bioactive Ingredients	Responsible Compound/Ingredient	Physiological Function Mechanism
Antioxidant effect	Proanthocyanidins, anthocyanins, flavonoids	Reduce oxidative stress and protect cells from damage by scavenging free radicals and enhancing antioxidant enzyme activity.
Anti-inflammatory effect	Proanthocyanidins, anthocyanins	Inhibit the production of inflammatory mediators, e.g., reducing the production of prostaglandin E2 and proinflammatory cytokines (such as TNF-α, IL-6).
Cardiovascular protection	Proanthocyanidins, anthocyanins	It has a protective effect on the cardiovascular system by improving vasodilation, lowering blood pressure, reducing blood clot formation, and reducing LDL oxidation.
Anticancer effect	Proanthocyanidins, anthocyanins	Induces cancer cell apoptosis, inhibits tumor growth and metastasis, and enhances the effect of chemotherapy drugs.
Antidiabetic effect	Proanthocyanidins, anthocyanins	It is beneficial against diabetes through mechanisms such as improving insulin sensitivity, promoting glucose uptake, and lowering blood sugar levels.
Antibacterial effect	Proanthocyanidins, anthocyanins	Directly combats bacteria or inhibits bacterial growth through mechanisms such as affecting bacterial cell walls and membrane structures.
Antiviral effect	Proanthocyanidins, anthocyanins	Interferes with the virus replication process, including inhibiting virus adsorption and invading host cells or interfering with virus replication.

## 4. *Aronia melanocarpa’s* Development Status

### 4.1. Aronia melanocarpa Juice

**Figure 5:**
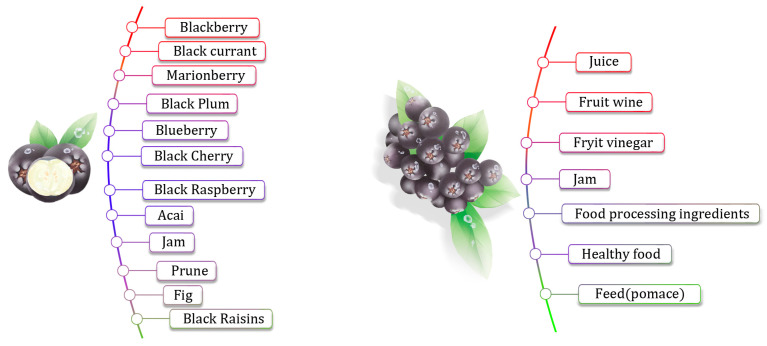
Various uses of *Aronia melanocarpa* fruit.

Because *Aronia melanocarpa* fruit contains polyphenols, its fresh fruit and pure juice have a poor taste [82]. This has a negative impact on its product development. The fruit can be deeply processed and can be made into dried fruit, fruit powder, juice, fruit wine, etc., as shown in Figure 5. Zhang et al. [83] used Lactobacillus plantarum C8-1 to ferment it and developed an *Aronia melanocarpa* fermented beverage with nutritional value and a unique fruity flavor. Cui et al. [84] used apples and *Aronia melanocarpa* to develop a compound juice with higher biological activity. Wang et al. [85] used biological sugar-lowering technology to reduce the sugar content of fruit juice to less than 1%. Then, they used *Aronia melanocarpa* to make low-sugar juice. This juice product has a low sugar content, a sweet and sour taste, and meets the needs of modern consumers.

### 4.2. Aronia melanocarpa Fruit Wine

With the rise of post-1990s and postmillennial groups, the main body of alcohol consumers is slowly changing. Traditional liquor, rice wine, beer, and other categories can no longer meet the needs of these people in terms of taste, but low-alcohol fruit wine products are welcomed by consumers in the market. Han et al. [86] invented a method for brewing *Aronia melanocarpa* fruit wine. Through juice preparation, fermentation, aging, post-ripening, purification, filtration, sterilization, and other processes, a fruit wine with a pleasant aroma, unique flavor, and refreshing taste is obtained. Yang et al. [87] developed an *Aronia melanocarpa* fermented wine, and the phenolics in *Aronia melanocarpa* can be well preserved in the wine. In addition, Gao [88] compared the traditional brewing method and the CO_2_ maceration method and found that the alcohol content of the dry wine obtained by the CO_2_ maceration method was higher. The total acid and tannins are lower, and the type, quantity, and sensory evaluation of aroma substances are better.

### 4.3. Aronia melanocarpa Fruit Vinegar

As a health drink, fruit vinegar can help improve human immune function and maintain human health. It is especially suitable for the elderly, women, and children to drink and has great market prospects [89]. *Aronia melanocarpa* is fermented into a vinegar product, which not only has the effects of antioxidants and preventing cardiovascular and cerebrovascular diseases but can also be used as a daily drink for young people. Wang [90] invented a 100% fermented *Aronia melanocarpa* vinegar drink which directly uses *Aronia melanocarpa* fruit as a raw material. It is obtained through pretreatment; the juice is produced using lactic acid fermentation, alcoholic fermentation, acetic acid fermentation, filtration, sterilization, blending, and filling. There is no need to add water or dilute the original vinegar during the entire production process, and the resulting fruit vinegar beverage product has a good taste.

### 4.4. Aronia melanocarpa Jam

With the improvement in living standards, people’s eating patterns have also changed. Breakfast has gradually changed from millet and dough sticks to bread, eggs, and milk, and jam has found itself on the family table of ordinary people. Wang et al. [91,92] used white wine and fruit vinegar to remove astringency from *Aronia melanocarpa*. After blanching, crushing, grinding, and then supplementing it with ingredients such as citric acid, sugar, and xanthan gum, a sweet and sour jam product was made through processes including cooking, concentration, canning, sterilization, cooling, and packaging.

### 4.5. Food Processing Ingredients

*Aronia melanocarpa* can be added as a food ingredient. It is not only safe and reliable, but also possesses nutritional value and physiological properties. Yoon et al. [93] used *Aronia melanocarpa* fruit powder as raw material to make bread. It was found that as the fruit powder content increased, the pH of the bread decreased, and the acidity increased. The color of the bread also changed: the bread with 10% *Aronia melanocarpa* fruit powder added appeared reddish. The bread had a longer shelf life than the control. Kaack et al. [94] used *Aronia melanocarpa* as raw material that was separated, extracted, and concentrated to produce food colorants. Research has found that *Aronia melanocarpa* anthocyanins are more stable than strawberry and black currant anthocyanins. When 10% *Aronia melanocarpa* juice is used as a coloring agent for processing plum juice, satisfactory color and a better flavor can be obtained.

### 4.6. Health Food

**Figure 6:**
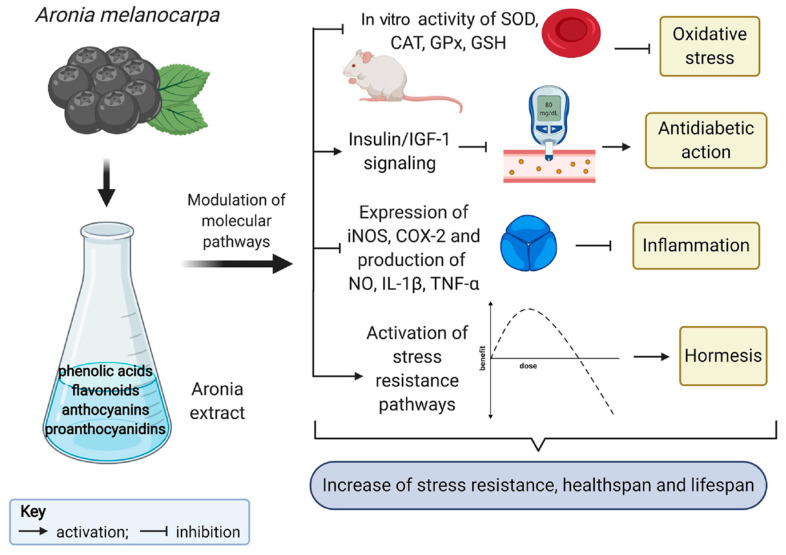
Antiaging effects of *Aronia melanocarpa* [100].

Since the extracted *Aronia melanocarpa* polyphenols are very sensitive to external factors, such as light and heat, *Aronia melanocarpa* capsule technology needs to be developed in depth. Tang [95] used microcapsule technology to control the release of anthocyanins, extending the storage period of anthocyanins and enhancing the stability of anthocyanins. Wen [96] modified *Aronia melanocarpa* pomace to improve the extraction rate of soluble dietary fiber (SDF) and then conducted research on the production process of *Aronia melanocarpa* pomace SDF tablets. This product can prevent rectal cancer, constipation, and hemorrhoids and produces a feeling of fullness after consumption, so it can be developed as a weight loss food. Wang et al. [97] prepared effervescent granules of *Aronia melanocarpa* fruit, and testing showed that the anthocyanin content in the product was as high as 96.43 mg/g. Teng [98] carried out research on the production process of *Aronia melanocarpa* chewable tablets. *Aronia melanocarpa* freeze-dried powder was mixed with proportions of mannitol, starch, aspartame, microcrystalline cellulose, and micronized silica gel and compressed into tablets. Testing found 28.26 mg of proanthocyanidins per chewable tablet. He [99] invented a complex nutrient for preventing and treating hypertension and hyperlipidemia. It contains *Aronia melanocarpa*, hawthorn, notoginseng flower, golden camellia, corn silk, bitter melon, cottonseed oligosaccharide, egg white ferment, ginseng baking powder, wheat oligopeptide, olive fruit powder, and grape seed extract. *Aronia melanocarpa* may have antiaging effects on the human body through stimulant effects, activation of antioxidant defenses, modulation of insulin/insulin-like growth factor 1 (*IGF-1*) signaling, and anti-inflammatory activity [100], as shown in Figure 6.

## 5. *Aronia melanocarpa* Juice Processing Technology

Raw juice is obtained from fruits and vegetables through simple processes such as crushing, pressing, and coarse filtration. It is then processed on the basis of the original juice to obtain a variety of products such as clear juice, turbid juice, concentrated juice, not-from-concentrate (NFC) juice, and juice powder [101]. In addition, because different fruit and vegetable juice products have different forms, they have their own characteristics in processing technology. For example, clear juices need to be clarified and filtered, turbid juices need to be homogenized and degassed, and concentrated juices need to be concentrated and dehydrated [102]. However, during juice manufacturing and product processing, various factors, such as oxygen, temperature, light, and metal ions, may affect the active ingredients in juice, thereby reducing the nutritional value and sensory quality of the juice [103]. Therefore, it is of great significance to ensure the quality of juice products by measuring the changes in their main components during the processing of juice products and selecting appropriate processing conditions.

### 5.1. Juice Clarification Technology

The general requirements for clarified juice refer to a clear and transparent liquid appearance without obvious sediments, suspended solids, non-discoloration, and visible impurities [104]. Therefore, clarification, on the one hand, is carried out to remove the suspended elements or sediment produced during the juicing process. On the other hand, macromolecular substances, such as starch and pectin, that are dissolved in the original fruit and vegetable juice and cause precipitation or turbidity need to be removed [105] in order to truly and effectively extend the shelf life of the product.

#### 5.1.1. Clarification Using a Clarifying Agent

Clarifying refers to the process of adding a particular substance (a clarifying agent) to the fruit and vegetable juice system so that it interacts with the components that cause turbidity in the system and forms a precipitation. This method achieves clarification by removing precipitates by filtration or centrifugation. Clarifying agents can be used alone or in combination with enzyme preparations. Depending on the characteristics of different juices, the ideal clarification effect can be obtained. There are many types of clarifiers; commonly used ones include gelatin, chitosan, diatomaceous earth, polyvinylpyrrolidone (PVPP), pentonite, activated carbon, and resin. Gelatin, tannins, silica sol, and other substances interact with pectin, polyphenols, proteins, etc., in fruit and vegetable juices to form large particles which can be separated by differential centrifugation, filtration, and other methods [106]. Activated carbon, PVPP, etc., can remove colloidal substances, pigments, and other substances in juice through physical adsorption or hydroxyl complexing ability [107].

The principle behind chitosan clarification is that it can form a stable colloidal structure in fruit and vegetable juices by interacting with negatively charged proteins, pectin, and other substances [108]. Due to its advantages of nontoxicity, fast clarification speed, and low price, it has been increasingly used in the clarification of fruit and vegetable juices in recent years. Jie et al. [109] used chitosan as a clarifier to clarify compound fruit and vegetable juices using celery, tomato, and Chinese cabbage as raw materials, and the clarification effect was good. Liu et al. [110] conducted a comparative study on the effects of gelatin and chitosan on the clarity of Schisandra fruit juice drinks, using gelatin and chitosan as clarifiers, and found that chitosan was more effective than gelatin.

#### 5.1.2. Enzymatic Clarification

Enzyme treatment generally refers to the use of enzyme preparations, usually including pectinase, cellulase, hemicellulase, etc., to clarify fruit and vegetable juices. In the process of fruit and vegetable juice processing, enzyme treatment can achieve the purpose of increasing the juice yield, improving clarity, and extending the storage period of fruit and vegetable juice without losing the nutrients of the juice [111]. The principle behind pectinase clarification of fruit and vegetable juice is the hydrolyzation of pectin substances. The positively charged protein particles initially wrapped inside are exposed due to partial hydrolysis of pectin. Other negatively charged particles collide with it, causing flocculation and precipitation. During the precipitation process, the floc will absorb other suspended solids in the fruit and vegetable juice. Finally, clarification is achieved through centrifugation and filtration. Commercial pectinase preparations generally contain different enzyme activities, such as cellulase, hemicellulase, etc., which can synergize with pectinase to completely degrade pectin. At present, enzymatic clarification has been widely used in various juices such as blueberry juice and pear juice. Shi et al. [112] also obtained good clarification effects when using the enzymatic method to clarify a ginkgo beverage.

#### 5.1.3. Membrane Clarification

The principle behind membrane clarification is the use of ultrafiltration or microfiltration membranes with different molecular weight cutoffs to remove precursor substances in juice that are prone to turbidity and secondary precipitation, such as proteins, starches, polysaccharides, and tannins [113]. On the one hand, the use of membrane clarification can simplify the traditional fruit and vegetable juice clarification process steps and reduce the consumption of enzyme preparations. On the other hand, it can be used at lower temperatures so that the original flavor and nutrients of fruit and vegetable juices are better retained.

At present, research on membrane clarification mainly includes process parameters such as the membrane type, molecular weight cutoff, operating pressure, and feed liquid flow rate. However, ultrafiltration has the disadvantages of complex process operations and high membrane prices [114]. Qian et al. [115] clarified longan juice through the membrane method and found that the obtained longan juice was clear and transparent, and the nutrients were not lost to a great degree. Gong et al. [116] found that membrane clarification can also achieve better clarification effects when producing orange juice.

### 5.2. Turbid Juice Homogenization Technology

A characteristic of turbid juice is that, after the fruits and vegetables are pulped or crushed, they contain their own particulate matter and are turbid and uniform. Compared with clear juices, cloudy juices have higher nutrients and soluble solids content. However, the stability time of cloudy juice in its natural state is short. The solid particles in the turbid juice are of different sizes and unevenly distributed, resulting in rapid precipitation. As time elapses, the upper liquid gradually turns into a clear liquid, which easily produces the impression in consumers that the product is old or dysfunctional [117,118]. Therefore, how to keep the turbid juice stable and non-deteriorating while maintaining its color and flavor has always been a focus of research.

Traditional thermal sterilization technologies include pasteurization, high-temperature instant sterilization, etc., which inactivate enzyme activity and sterilize through high temperatures. However, high temperatures often lead to the loss of food nutrients, flavor, and color, making it difficult to ensure food safety and quality. Therefore, nonthermal processing has become the current trend in fruit and vegetable juice processing. High-pressure homogenization technology (HPH) has also gradually come into people’s field of vision. Studies have shown that HPH can maintain the original color, aroma, and taste of fruit and vegetable juices and homogenize the turbid juice to make the particles more refined and evenly distributed, improving stability [119]. High-pressure homogenization technology is often used industrially in the 20–50 MPa range to produce stable beverages, preventing harmful changes caused by thermal processing. The biggest advantage of cloudy juice is that it simultaneously preserves microorganisms and improves stability, extending the shelf life of the product. The principle behind the production of cloudy juice is that pressurized liquid is forced through a gap (or interruption valve) a few microns wide. The resulting pressure drop simultaneously produces strong fluid–mechanical stresses, such as local cavitation, shear stress, collision, and turbulence, thereby breaking large particles into smaller particles and improving the turbidity stability of fruit and vegetable juices [120].

#### 5.2.1. Effect of Homogenization on Stability

An obvious advantage of using high-pressure homogenization technology in the production and processing of fruit and vegetable juices is that it can reduce the particle size of fruit and vegetable juices and increase the viscosity of fruit and vegetable juices. Chandi et al. [121] found that pasteurized citrus juice showed a bimodal particle size distribution when observing the particle size distribution of fruit and vegetable juices through a laser particle size analyzer. After high-pressure homogenization treatment, the distribution is unimodal, with a smaller particle size and narrower distribution range. This is consistent with the conclusion of the homogenized apple–kiwi mixed juice, indicating that high-pressure homogenization can indeed refine the particle size in fruit and vegetable juices and evenly distribute them. The apparent viscosity and dynamic yield stress values of homogenized juice also gradually increased with the increase in homogenization pressure. This is because high-pressure homogenization treatment causes cell division and fragmentation of fruit and vegetable juices, which not only increases the surface area of suspended particles but also releases cell wall components, such as pectin and protein, into the clear liquid of the juice [122]. High-pressure homogenization may enhance interparticle interactions through van der Waals forces, electrostatic forces, and hydration forces. However, since the rheological properties of juice under high-pressure homogenization also exhibit progressive behavior, excessive pressure has little effect on improving the viscosity of juice.

#### 5.2.2. Effect of Homogenization on Physical and Chemical Properties

Relevant studies have shown [123,124] that high-pressure homogenization has no significant effect on the pH value, soluble solids, and organic acid content of juice. However, it will lead to a reduction in titratable acid content and a change in the color of the juice. After high-pressure homogenization treatment, the L* value (brightness) and b* value (yellow) of orange juice and apple juice were higher, and the a* value (red) did not significantly change. The L* value changes because its size is positively correlated with the turbidity of the juice and negatively correlated with the D[3,4] and D[2,3] values. Since the ultra-high-pressure homogenization (UHPH) treatment reduces the particle size of the juice, its brightness increases. It has been reported [125] that as the homogenization pressure increases, the tomato pulp becomes clearer, and the red and yellow colors become more saturated. High-pressure homogenization destroys the cells of tomato pulp and the carotenoid–protein complex in the chromoblasts, causing lycopene to leak. However, some studies have shown that homogenization pressure and processing times have no significant effect on the color of juice.

#### 5.2.3. Effect of Homogenization on Active Ingredients

The nutrients in fruit juice mainly include vitamin C, polyphenols, flavonoids, anthocyanins, carotenoids, etc. Daily drinking of fruit juice is of great help in supplementing nutrients and improving human health. Relevant research by Tribst et al. [126] showed that after high-pressure homogenization treatment, the Vc content of citrus juice will decrease slightly (by 5%). As the pressure increases, the loss rate increases. Significant changes in flavonoid content and high bioavailability occur without pasteurization. Similarly, Shuai et al. [127] showed that the total phenolics, total flavonoids, and total anthocyanins contents of mulberries treated with 160 MPa high-pressure homogenization were reduced by 39.91%, 27.35%, and 24.41%, respectively.

It is known that the presence of enzymes and oxygen, which increases pH and temperature, leads not only to the degradation of anthocyanins but also to their polymerization. High-pressure homogenization increases the temperature of liquid, so there are indications that high-pressure homogenization and ultra-high-pressure homogenization may lead to a decrease in anthocyanin content. However, some studies have shown that during the treatment process, an almost constant anthocyanin concentration was obtained under a pressure of 30~150 MPa [128,129]. The results indicate that high-pressure homogenization technology is a promising emerging method that can provide optimal quality encapsulation and stability of anthocyanin-rich emulsions.

**Figure 7:**
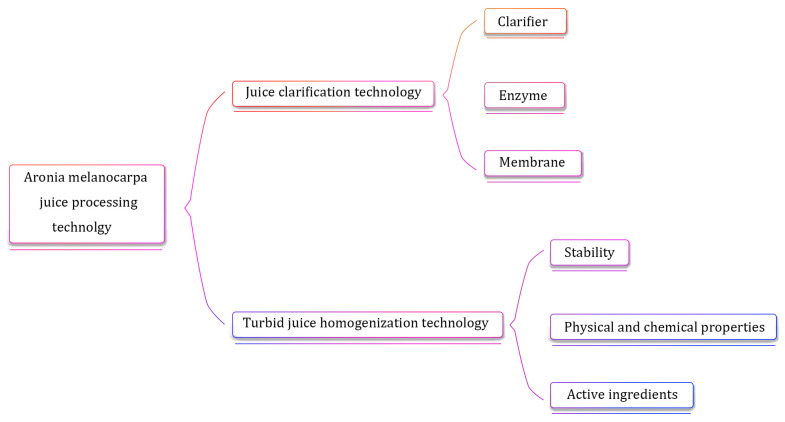
*Aronia melanocarpa* fruit processing technology and influencing factors.

As shown in Figure 7, the above shows that there are large differences in the impact of different processing technologies on the nutritional components of juice. Various factors need to be integrated to deeply analyze the causes and mechanisms of their formation, and individual parameters should be optimized for different juices.

The content of active ingredients in *Aronia melanocarpa* juice may vary greatly depending on the variety, maturity, growth conditions, and processing methods of the raw materials, which brings challenges to product standardization and quality control.

Despite the health benefits of *Aronia melanocarpa*, its naturally sour and astringent taste may affect acceptance by some consumers. Therefore, it is necessary to develop product formats with more favorable taste and flavor. Although studies have shown the health benefits of *Aronia melanocarpa*, more in-depth scientific research is needed to support its specific mechanism of action, optimal consumption, and safety and effects of long-term intake. The production cost of high-quality *Aronia melanocarpa* juice is relatively high, which may limit its accessibility and popularity in some markets. These problems restrict the comprehensive development of *Aronia melanocarpa* juice in the field of food processing.

As consumer demand for healthy food increases, *Aronia melanocarpa* juice production and processing technology continues to evolve. In order to maximize the retention of antioxidants and other nutrients in *Aronia melanocarpa*, research and application of mild processing techniques will become a trend. This includes techniques such as cryogenic concentration, ultrasound-assisted extraction, and high-pressure processing. *Aronia melanocarpa* juice can be combined with other ingredients with health benefits (such as other juices, prebiotic fibers, vitamins, and minerals) to develop a variety of functional beverage products to meet the specific health needs of the market. Of course, the by-products produced during the processing of *Aronia melanocarpa*, such as pomace, are rich in nutrients and can be used to develop other types of food and beverages, such as fiber supplements, breads, and cookies. This helps improve the economics and sustainability of overall production. Therefore, the corresponding supporting production chain needs to be considered.

As consumers become more concerned about the environment and health, *Aronia melanocarpa* produced using sustainable agricultural practices, as well as being certified organic, will become more popular. Therefore, how to use scientific and healthy methods to meet consumers’ high-standard dietary needs during the processing of *Aronia melanocarpa* juice is also a focus that needs to be considered in the future.

## 6. Current Status of Utilization of *Aronia melanocarpa* Pomace in Feed

As the main by-product of the fruit processing industry, pomace cannot be simply treated as waste. According to a large number of studies, pomace is also rich in nutrients and can provide protein, fat, and a variety of bioactive compounds. However, due to insufficient understanding of the bioactive components and properties of fruit waste, and because the further processing of pomace often requires higher investment costs, the development and utilization of pomace is still insufficient.

A large number of studies have found that the pomace produced during the deep processing of fruits still contains a large amount of nutrients such as protein, fat [130], and bioactive compounds [131]. *Aronia melanocarpa* pomace (AMP) is the main by-product produced during the intensive processing of its fruit. During the physical squeezing of *Aronia melanocarpa*, a large amount of juice flows out, and the remaining pomace is mainly composed of four parts: pulp, peel, seeds, and a small amount of stems accounting for about 16% [132]. The main nutritional components of *Aronia melanocarpa* pomace vary greatly depending on the fruit tree species, fruit maturity, production, and processing methods and the production requirements of different manufacturers.

Although the composition of *Aronia melanocarpa* pomace differs, during the pressing process of the fruit, its chemical composition is not changed. Its main components remain pulp, peel, seeds, and a small quantity of stems. Because a large amount of phenolic substances in its peel and pulp cannot be dissolved in water, it is difficult for the components to flow out with pressing. Therefore, the pomace still contains a large number of functional ingredients [132], such as anthocyanins, proanthocyanidins, phenolic acids, and other bioactive ingredients, which also have high anti-inflammatory and antioxidant activities.

**Figure 8:**
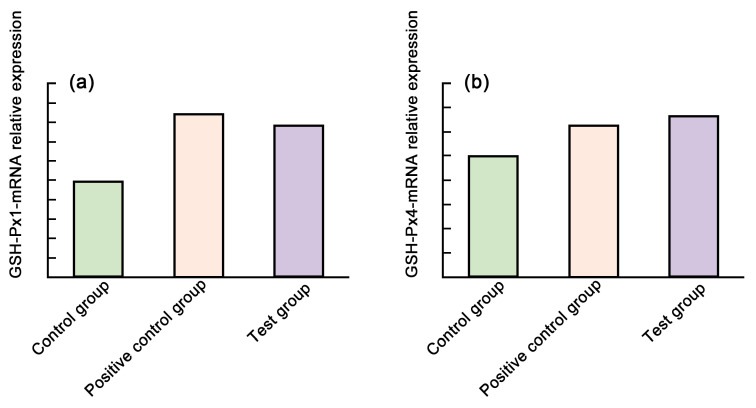
Effect of *Aronia melanocarpa* pomace on the relative expression of (**a**) GSH-Px1 and (**b**) GSH-Px4 mRNA in liver [133].

Ju et al. [133] added 0.2% *Aronia melanocarpa* pomace to piglet feed and continued feeding for 28 days. The results showed that adding AMP to feed not only increased the daily feed intake of piglets but also reduced the diarrhea rate of piglets and increased the gene expression of GSH-Px1 in the liver. It also increased the GSH-Px4 (glutathione peroxidase) content in the serum, as shown in Figure 8. This shows that *Aronia melanocarpa* pomace exerts the fruit’s excellent antioxidant function. Lipinska et al. [60] selected 24 Polish Merino lambs and fed them AMP at 150 or 300 g/kg according to the lamb’s weight. After 90 days of continuous feeding, it was found that there was no adverse effect on lamb health, which provides a theoretical reference for the application of AMP in animal feeding.

The above results indicate that using *Aronia melanocarpa* pomace (AMP) for making animal feed is a good choice. Adding an appropriate amount of AMP to pig feed can not only reduce production and breeding costs and improve economic benefits but also solve the problem of pomace processing and reduce environmental pressure. At the same time, it alleviates the dilemma of a shortage of feed resources. There are currently few studies on the application of AMP in livestock and poultry breeding. However, because it is rich in bioactive substances, such as proanthocyanidins and flavonols, *Aronia melanocarpa* pomace has great research value and development potential.

## 7. Summary and Outlook

*Aronia melanocarpa* is an excellent forest tree species with edible, medicinal, economic, ornamental, and ecological value and has extremely broad application prospects. International research on the development of *Aronia melanocarpa* fruit-related products is also very extensive. It has expanded from the initial food and medicine to health care products, food additives, food color packaging, etc., showing great potential in many fields. At present, the demand for raw materials from deep-processing enterprises in Japan, South Korea, and other countries has surged and the supply has shown a trend of exceeding demand. Whether it is planting, exporting, product development, etc., *Aronia melanocarpa* fruit has excellent application and market prospects.

At present, due to the long history of cultivation and consumption and relatively complete basic research, the focus of international research on *Aronia melanocarpa* has gradually changed from basic research, such as analytical method development, chemical composition analysis, product process optimization, etc., to the following: ① the development of different types of food and the application of new processes (for example, dietary supplements prepared from juice rich in glucomannan fiber can be used to improve the dietary intake of obese people); ② new fields and new applications (for example, active ingredients with antioxidant, antidiabetic, and other activities can be used as co-pigments in the development of functional foods and as facial cream additives to improve skin aging); ③ the improvement of *Aronia melanocarpa* itself. For example, for the astringent taste caused by being rich in polyphenols, various polysaccharides can be added to improve its taste, or its metabolism and products can be studied to solve the problem of the low bioavailability of polyphenols such as anthocyanins.

Limited by the short introduction time and practical application time, Chinese research is still in its infancy, with the following three main shortcomings: ① lack of product evaluation and management (China does not yet have specific quality evaluation standards for *Aronia melanocarpa* fruits, nor has it established a quality evaluation system for its related products); ② product research and development needs further development (the research and development of related products is still in its infancy, with single product categories and application fields); ③ traditional extraction and purification processes. There is also insufficient understanding of the nutritional value and medicinal value of *Aronia melanocarpa* fruit. Deepening the understanding of the characteristics of *Aronia melanocarpa* fruit will provide a theoretical basis for developing new processes, new products, and new applications using it as raw material, providing full play to its advantages as a “new food raw material”, and providing assistance to promote the development and prosperity of China’s *Aronia melanocarpa* (“young berry”) industry.

Therefore, the subsequent research and development of *Aronia melanocarpa* in China can be based on the optimization of existing deep processing technology and active substance extraction methods and further research and understanding of its interactions with other compounds. The results can be used to understand the activity and bioavailability of *Aronia melanocarpa* components, establish recommended intake levels, and develop newer and more advanced processing technologies, allowing us to maximize and rationally process and utilize the active ingredients in the fruit, juice, and pomace of *Aronia melanocarpa* to determine its mechanism of action, safety, and effectiveness. At the same time, we can also develop a series of *Aronia melanocarpa* processing products to achieve factory, scale, and enterprise development. Even though processed *Aronia melanocarpa* products have good taste and flavor, they can also provide daily nutritionally active ingredients.

Proanthocyanidin B2, which is most abundant in pomace, has been proven to have significant anti-inflammatory, antioxidant, and anticancer effects. Due to the special properties of pomace, adding AMP to feed can increase the nutritional value of feed. Furthermore, the rich bioactive components in pomace can promote the health of animals by regulating the abundance of animal intestinal flora. AMP has a large output, mature extraction technology, and relatively low cost. Using it as a raw material in feed has been carried out rationally and solves the problem of processing pomace, leading to a reduction in production and breeding costs. At the same time, the use of AMP is of great significance to further alleviate the conflict between humans and animals competing for food, and it is important to explore and apply unconventional feed resources.

## Figures and Tables

**Table 1:** Main polyphenol components of *Aronia melanocarpa* [25,26].

Element	Mass Contents (μg/g)
Proanthocyanidin B1	129.78
Proanthocyanidins B2	3834.99
Proanthocyanidin B4	196.02
Rutin	134.40
Quercetin	79.10

**Table 2:** Comparison of polysaccharide content in some small berries [37].

Variety	Polysaccharide Contents (mg/g)
Wild blueberries	79.86
viburnum	63.68
Hawthorn	122.27
Shanjingzi	163.62
*Aronia melanocarpa*	140.82

## Data Availability

The data that support the findings of this study are available from the corresponding author upon reasonable request.
